# Deguelin Action Involves c-Met and EGFR Signaling Pathways in Triple Negative Breast Cancer Cells

**DOI:** 10.1371/journal.pone.0065113

**Published:** 2013-06-10

**Authors:** Rajeshwari Mehta, Harshadadevi Katta, Fatouma Alimirah, Rutulkumar Patel, Genoveva Murillo, Xinjian Peng, Miguel Muzzio, Rajendra G. Mehta

**Affiliations:** Cancer Biology and Analytical Chemistry Divisions, IIT Research Institute, Chicago, Illinois, United States of America; University of Medicine and Dentistry of New Jersey, United States of America

## Abstract

**Background:**

Treatment of breast cancer patients with antiestrogens and aromatase inhibitor(s) or Herceptin have shown significant success in steroid receptor positive or Her-2+ breast cancers respectively. However, choice of treatments for breast cancer patients with negative status for estrogen, progesterone receptors and HER2/neu is limited. As a result, search for appropriate therapy regimen for these triple negative breast cancers (TNBC) has become a major focus of investigations for many laboratories. Recently, Deguelin, a natural product isolated from African plant *Mundulea sericea* (Leguminossae) has shown both antiproliferative actions in various cancers including breast as well as chemoprenventive activity against carcinogen induced experimental cancers. In this report we evaluated efficacy and mechanism of action of Deguelin in triple negative breast cancer cell lines.

**Methods/Findings:**

In vitro, Deguelin in a dose and time dependent manner inhibited the growth of MDA-MB-231, MDA-MB-468, BT-549 and BT-20 cells. Deguelin (2 or 4 mg/kg body weight), when injected intraperitoneally, reduced the in vivo tumor growth of MDA-MB-231 cells transplanted subcutaneously in athymic mice. Moreover it was nontoxic as evident from daily observations on mobility, food and water consumption and comparison of bodyweight and other visceral organ weights with those in control animals at the termination of the study. The western blot analyses and immunostaining studies indicated that the deguelin effects may be mediated through EGFR-PAKT/c-Met p-ERK and NF-κB by down regulating their downstream targets such as p-STAT3, c-Myc, Survivin.

**Conclusion/Significance:**

These results suggest that Deguelin may have a significant therapeutic value for the treatment of TNBC patients.

## Introduction

Breast cancer is a heterogeneous group of diseases. Approximately 60–70% of breast cancers express estrogen receptors (ER) and/or progesterone receptors (PR). About 20–30% of breast cancers have amplified levels of human epidermal growth factor receptor (HER)2 protein. Treatment options, which inhibit the estrogen pathway or target amplified HER-2 are effective in the treatment of breast cancer in patients whose tumors express these targets. However, in approximately 15–20% of patients with breast cancer, the tumors do not express ER or PR and do not have amplification of HER-2 [1], [2]. These tumors are called triple-negative breast cancer (TNBC). Triple-negative breast cancers (TNBCs) are highly aggressive histological subtype, patients with TNBCs have very poor prognosis following progression after standard chemotherapeutic regimens. It is interesting to note that many selective signaling pathways (ERK, p-AKT) are highly activated due to specific gene overexpression such as EGFR [3], [4] in highly aggressive breast cancers. The triple negative breast cancers develop at an early age and are more frequent in premenopausal African American or Hispanic women [5], [6] as compared to other ethnic groups. While anthracyclin and texane based standard therapies have been shown to be effective initially, majority of patients show disease relapse eventually [7]. This clearly indicates that it is critical to identify selectively targeted agents for the management of TNBC. Recently we have shown that synthetic Deguelin, a compound present in an African plant, *Mundulea sericea,* inhibited in vivo growth of various breast cancer cells [8]. In this study we further explored the therapeutic efficacy of Deguelin on in vitro as well as in vivo growth of triple negative breast cancers.

By definition, TNBCs do not express steroid hormone receptors and Her-2. However overexpression of other growth factor receptors such as EGFR and c-Met is frequently reported for this subset of cancers [9], [10]. Typically, signaling through both EGFR and c-Met promotes cell survival and proliferation and is required by the cancer cells. In the present study, we hypothesized that deguelin interferes with the EGFR and c-Met signaling and this may in turn provide targeted therapy for patients with TNBC. In this report we evaluated efficacy of deguelin in TNBC cells both in vitro and in vivo. We also determined the possible mechanism of action of Deguelin in regulating expression of growth factor receptor signaling molecules.

## Materials and Methods

All in vivo studies were carried out according to the approved protocol by IIT Research Institutes Institutional Animal Care and Use Committee. Every effort was made to make sure that the studies were carried out humanely without causing any pain to the animals.

### Cell Culture

Human breast cancer cell lines MDA-MB-231, MDA-MB-468, BT-20 and BT-549 were obtained from American Type Culture Collection (Rockville, MD) and cultured as monolayers in MEM-E supplemented with 10% heat-inactivated fetal bovine serum (FBS), 100 µg/mL penicillin and 100 µg/mL streptomycin (Invitrogen™ by Life Technologies Grand Island, NY) and maintained at 37°C in a 5% CO2 atmosphere and 95%air.

### Chemicals

Deguelin was purchased from Sigma (Sigma Aldrich, St. Lois MO). For in Vitro studies it was dissolved in a 100% EtOH at concentration of 10 mM as stock solution. The stock solution was further diluted to 1 mM with absolute alcohol as working solution. For in vivo experiments, Deguelin was administered to the athymic mice in the form of a suspension in solutol/PBS solution (0.4% Solutol in PBS). Solutol was purchased from Sigma Aldrich, St Louis, MO. In order to make a suspension Deguelin was weighed, mixed with melted Solutol and then mixed with PBS. Inhibitors for ERK (UO126) and PI-3K/Akt (LY294002) were obtained from Santa Cruz Biotechnology, Santa Cruz, and dissolved in DMSO.

### Effect of Deguelin on Cell Proliferation in vitro

Breast cancer cells were incubated with increasing concentration of Deguelin ranging from 31 nM to 500 nM for 24, 48 and 72 h. At the termination the cells were trypsinized and cell proliferation was evaluated by counting cells using Z-series Coulter counter (Beckman Inc. Brea, CA). Data are presented as Mean±SE percent of control (vehicle treatment only).

### Immunofluorescence Assay

Cells cultured on glass coverslips were treated with vehicle or Deguelin containing media. At the end of incubation cells were rinsed with PBS, fixed in 5% buffered formalin permeabilized with ice cold methanol and treated with 5% BSA in PBS at RT for 5 min to block nonspecific binding. Cells were then incubated with primary antibody against specific antigen (diluted in PBS containing 1.0% BSA according to the manufacturer’s instructions), washed extensively in PBS and treated with appropriate alexa 488 or alexa 568 labeled secondary antibodies (1∶40 dilution, Molecular Probes/Invitrogen (Eugene, OR). Cells were then washed with dH20, and mounted in media containing DAPI/PI. Immunostaining was examined using fluorescence microscope (Olympus) equipped with appropriate filters for alexa488, alexa568 and DAPI/PI. Representative images captured at 40× objectives are shown.

### Immunohistochemistry

Cells cultured on glass coverslips were fixed in 4% formalin (10 min), washed with phosphate buffer saline (PBS) and permeabilized in ice cold 100% methanol for 3 min. To block non- specific immunostaining, cells were incubated with 1% BSA in PBS for 30 min followed by 1 h incubation with appropriate specific primary antibodies. For immunohistological staining of tumors and lung metastasis, paraffin embedded tissue sections were deparaffinized and rehydrated by passing through a decreasing series of alcohol. Tissue sections were rinsed in PBS and then antigen retrieval was performed using citrate buffer. Nonspecific staining was blocked by incubating tissue sections in 5% nonfat dry milk/BSA. The sections were incubated with primary antibodies (cells for 24 h) in a humidified chamber. All antibodies were diluted according to manufacturer’s instructions in PBS containing 1.0% BSA. All primary antibodies (c-Met, p-ERK, IL-6, PCNA, ERK ) were obtained from Santa Cruz Biotechnology, Santa Cruz, Burlingame, CA, antibodies against NF-κB (ser 276), p-AKT, c-Myc, Survivin, phospho Tyrosine 705 STAT3 and STAT3 were obtained from Cell signaling Technology, Danvers, MA. After incubation with primary antibody, cells or tumor sections were rinsed extensively with PBS and then treated with biotinylated appropriate antibody followed by Biotinylated horseradish peroxidase macromolecular Complex using ABC Vectastain kit (Vector Laboratories, Burlingame, CA). Immunoreactivity to specific protein was visualized using DAB (3, 3′ Diaminobenzidine, Vector Laboratories, Burlingame, CA) as a chromogen. In some cases immunostaining was enhanced using NiCl2 with DAB. Cells/tumors were counterstained with hematoxylin, dehydrated and cleared in xylene and then mounted using permount. All images were captured at 20–40× magnification using Olympus microscope. .

### Western Blot Analysis

Western Blot analyses were conducted as described previously [8]. Briefly, 1×10^6^ cells were treated with Deguelin/vehicle only or other drugs as indicated. Total protein was extracted using RIPA buffer (Santa Cruz Biotech, Santa Cruz, CA). The protein lysates (75 µg) were electrophoresed on a 10% SDS gel and transferred to Immnobilon-P (PVDF, pore size 0.45 µm; Millipore, Bedford, MA) membranes. Membranes were subsequently rinsed with PBS and blocked with 5% non-fat dry milk in TBST (10 mM Tris–HCl, 150 mM NaCl, 0.01% Tween-20) for 1–2 h at room temperature. The blots were incubated overnight at 4°C with appropriate primary antibodies. Following incubation, membrane was rinsed in TBST, and then incubated at room temperature with appropriate HRP labeled secondary antibody from (Santa Cruz Biotechnology) at room temperature.

### In Vivo Studies

Six to seven weeks old female athymic mice (nu/nu) were purchased from Charles River Laboratories (Wilmington, MA) and housed in a barrier free environment at IIT Research Institute Animal facility under 24±2°C temperature, 50±10% relative humidity, and 12-hour light/12-hour dark cycle. Mice were provided with sterile mouse chow and water ad libitum. This study was carried out in accordance with the recommendations in the Guide for the Care and Use of Laboratory Animals of the National Institutes of Health. All in vivo studies were carried out under an approved protocol by the IIT Research Institute’s Institutional Animal care and Use committee (IACUC). MDA-MB-231 cells (3.0 million cells/animal) were suspended in sterile PBS and then injected subcutaneously into the dorsal flank region using 23 g hypodermic needle. Animals were observed daily for the growth of palpable tumor at the site of injection. Once the tumor (approximately 50 mm^3^) appeared the mice were randomized in to three groups, animals receiving either 1) vehicle as a control 2) Deguelin treatment at 2 mg/kg bodyweight dose or 3) Deguelin at 4 mg/kg body weight. Each group consisted of 10 animals. Vehicle or Deguelin was administered through i.p. injection daily for 21 days. Animals were monitored daily for the signs of drug/vehicle associated toxicity and weighed once weekly. Growth of tumor at the site of cell injection was monitored every alternate day and of tumor size was measured using calipers. Tumor volume was calculated by using the well-established formula: tumor volume (mm3) = π/6 length×width×depth. Data represent the mean tumor volume+SE (mm^3^) in each group. The animals were sacrificed at the indicated time unless they appeared to be moribund or tumors showed sign of necrosis. At termination, the tumor was excised, freed from connective tissue and other organs, a small piece was fixed in 10% buffered formalin and remaining tumor was snap frozen for future biochemical analysis. Liver, lung, kidney and spleen were excised and weighed.

### Statistical Analyses

All experiments were performed in triplicates. Data generated were subjected to appropriate Statistical analysis using Graph Pad Prism 5.0 (Graph Pad Software, La Jolla, CA). Differences between means from two different groups were subjected to student’s ‘t’ test whereas one-way analysis of variance (ANOVA) followed by Bonferroni adjustment was used to analyze significant differences between three or more groups. In vivo growth data was subjected to paired ‘t’ test. Results were considered to be significantly different when p value was <.05.

## Results

### Effect of Deguelin on Cell Proliferation

**Figure 1:**
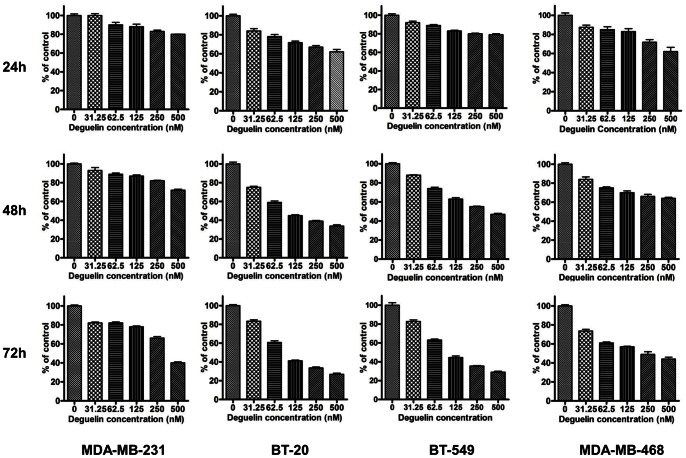
Effect of Deguelin on the growth of MDA-MB-231, BT-20, BT549 and MDA-MB-468. The cells were plated in six well plates, incubated at 37°C overnight and then treated with either vehicle only or Deguelin at indicated concentration. Number of cells in each well was counted using coulter counter. Number of cells in vehicle treated well was considered as 100%, data represent mean±SE (% of control) of at least three independent observations. * indicates significant difference between control and treatment.

Previously Deguelin was shown to have antiproliferative action in various cancers including breast cancer. In this study we examined the effects of Deguelin on four different triple negative breast cancer cells (MDA-MB-231, BT-20, MDA-MB-468, BT-549). Cells were incubated in the culture media containing varying concentration of Deguelin (0–500 nM) for 24–72 h. Deguelin significantly (p<0.05) inhibited the growth of all the cell lines studied in a dose and time dependent manner, maximum growth inhibition was noticed at 72 h with 500 nM Deguelin (Figure 1).

### Response of MDA-MB-231 Cells in the Presence of EGF

**Figure 2:**
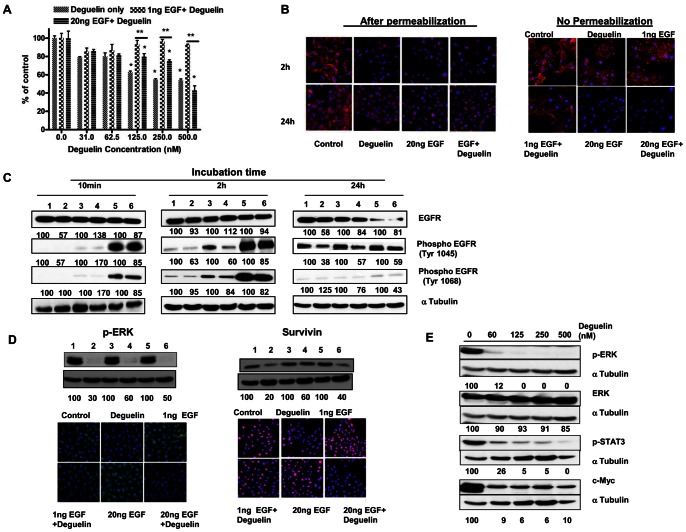
Effect of Deguelin on the growth and expression of signaling molecules in TNBC cells. A. Effect of deguelin on MDA-MB 231 cells in the presence of EGF (1 ng or 20 ng) alone, Deguelin alone (31–500 nM)or Deguelin+EGF. The cells were plated in six well plates, allowed to attach overnight, then treated with indicated test compound/(s) (in serum free media) and incubated for 72 h at 37°C. Number of cells was counted in each well using coulter counter. Data represent % cells in the respective control treatment. * indicates significant difference as compared to respective control, ** indicates significant difference (p<0.05) between the groups shown. B. Effect of Deguelin on total (after permeabilization)and cell surface (without permeabilization) EGFR expression in the presence/absence of indicated concentration of EGF. The cells plated on glass coverslips were incubated for 10 min-24 h at 37°C in the serum free media containing vehicle only, Deguelin (250 nM) or Deguelin+EGF as indicated. At the end of incubation cells were fixed in 4% buffered formalin, permeabilized and then processed for immunofluorescence examination of EGFR (red) and mounted in DAPI (blue) containing media. Representative photographs captured using fluorescence microscope equipped with appropriate filters are shown. Red =  EGFR expression, Blue =  DAPI staining of nuclei. Data are shown for 2 and 24 h post treatments. C. Effect of Deguelin, EGF and EGF+Deguelin on EGFR/1045-phospho EGFR and 1068-Phospho EGFR levels in MDA-MB-231 cells. MDA-MB-231 cells were treated for 10 min–24 h with indicated treatments in serum free media as described above. At the end of incubation cells lysates were prepared and subjected to western blot analysis. For loading control appropriate control proteins were developed using appropriate antibodies. Lanes 1 = control, 2 =  Deguelin 250 nM, 3 = EGF1 ng 4 =  EGF 1 ng +Deguelin 250 nM, 5 =  EGF 20 ng 6 = EGF20 ng+Deguelin 250 nM. Number under each lane indicate % of normalized (protein intensity/control protein intensity) specific band intensity in relation to control treatment. Control was considered as 100%. D. Western blot and Immunofluorescence analyses of p-ERK and Survivin in MDA-MB-231 cells treated for 24 h with vehicle only, Deguelin (250 nM) in the presence or absence of EGF. Serum free media was used for this study. Lane 1 =  Control, 2 =  Deguelin 250 nM, 3 = 1 ng EGF, 4 = 1 ngRGF+Deguelin250 nM, 5 = 20 ng EGF, 6 = 20 ng EGF+Deguelin. For western blot number under each band indicates % of intensity of normalized values as described above. In immunofluorescence images, green fluorescence indicates p-ERK, red immunofluorescence indicates Survivin and Blue staining is due to nuclear counterstaining with DAPI. Representative images are shown. E. Western blot analysis of various cell signaling molecules (p-ERK, ERK, p-STAT-3, c-Myc) in MDA-MB-231 cells treated for 24 h with varying concentration of Deguelin in serum free media. Intensity of specific band is normalized in relation to loading control protein intensity, ratio in vehicle treated control with housekeeping protein control was considered as 100%. Number under each band indicate % of control intensity.

EGF is a known mitogen to stimulating cell proliferation [11]. We examined the effect of Deguelin in the presence or absence of EGF on growth of MDA-MB-231 cells. Cells were incubated at 37°C in serum free media containing low (1 ng) or high (10 ng) concentration of EGF alone, Deguelin alone (250 nM) or Deguelin +EGF (1 or 10 ng) for 72 h. As expected Deguelin showed growth inhibition of MDA-MB-231 cells however EGF failed to show growth stimulation when deguelin was incubated in the presence of EGF. Interestingly, Deguelin at all concentrations failed to reduce cell numbers in the presence of 1 ng EGF but in the presence of EGF 20 ng reinstated deguelin mediated growth inhibition. Our results suggest that while deguelin inhibits growth of MDA MB231 cells, EGF has a differential role. At lower concentration it is protective against deguelin effect whereas at higher EGF concentration this protection is lost (Figure 2A).

### Effect of Deguelin on EGFR Expression

Majority of the TNBC cells show overexpression of EGFR [12]. The EGFR is activated by a family of growth factors namely EGF, Amphiregulin, TGFalpha and neuregulin [13]. Activated receptor induces signaling cascades mediated mainly through PI-3K- AKT Pathway. Deguelin has been shown to inhibit AKT activation [14], however the effect of Deguelin on EGFR is still not known. Therefore, we examined the effect of Deguelin on EGFR expression. MDA-MB-231 cells were incubated for various times ranging from 10-min-24 h in serum free media containing either Deguelin (250 nM), EGF (1 ng or 20 ng) or EGF (1 ng or 20 ng) +Deguelin. EGFR expression/localization was examined by immunofluorescence assay. In the cells treated with vehicle only, EGFR was mainly present on the cell membrane with some expression in the cytosol and nucleus after 10 min. of incubation. Following treatment with Deguelin no significant change in the intensity or localization of EGFR was noticed. Treatment with low dose of EGF (1 ng) EGFR expression pattern was also similar to that observed in controls. On the other hand cells treated with 20 ng EGF showed loss of cell surface expression of EGFR and peri-nuclear accumulation. EGF+Deguelin treatment showed EGFR expression pattern similar to those cells treated with EGF only (data not shown for 10 min).

At 2 h and 24 h post incubation, EGFR distribution in control cells was similar to that observed at 10 min, intense immunostaining was seen on the cell membrane. Intensity of cell surface EGFR expression was reduced after Deguelin treatment. In cells treated with 1 ng EGF, EGFR expression was similar to that observed in control, treatment of cells with Deguelin+EGF(1 ng) down regulated EGFR expression on the cell surface. In cells treated with 20 ng EGF some cell surface and in Deguelin+EGF(20 ng) treated cells only peri-nuclear EGFR expression was observed (Figure 2B).

We further confirmed that the effect of Deguelin is indeed on the expression of EGFR on the cell membrane. Cells were treated with EGF, Deguelin or EGF+ Deguelin in serum free culture media for 24 h. At the end of incubation cells were fixed in formalin and then processed for immunofluorescence without permeabilization process in order to observe only cell surface receptors. As shown in Figure 2B Deguelin treatment down regulated cell surface EGFR.

We aimed to examine whether reduced membrane associated EGFR expression observed at 2 h and 24 h also showed reduced site specific phosphorylation of tyrosine in the kinase domain responsible for the activation or ubiquitination of EGFR. Phosphorylated EGFR at tyrosine at position 1068 provides binding site for Grb2 protein whereas at tyrosine 1045 provides docking site for c-cbl protein and thus facilitates receptor degradation. We determined levels of total EGFR, phospho EGFR (Tyr1045, domain necessary for ubiquitination and phospho Tyr1068 GRB2 mediated signaling) in cells treated with Deguelin, EGF and EGF+Deguelin for 10 min to 24 h by western blot analyses. Total EGFR levels were not different at 10 min or 2 h following any treatment but clearly reduced in cells treated with Deguelin and EGF (20 ng) for 24 h as compared to control. Phospho tyrosin-1045 and 1068 specific EGFR protein bands were observed at 10 min and 2 h only when cells were incubated with EGF or EGF+Deguelin. Deguelin inhibited Phospho(Tyr1045) EGFR and to a lesser extent Phospho(Tyr1068) EGFR. Interestingly at 24 h, Phospho Tyr1045 EGFR expression was detected in all treatment groups, even in cells incubated in serum free media without any EGF. Reduced phospho Tyr1045 EGFR levels were observed in cells incubated with Deguelin alone or Deguelin+EGF (1 and 20 ng). A faint but distinct phospho Tyr1068 specific protein band was detected when cells were incubated with EGF (20 ng only), and was reduced in cells treated with Deguelin+EGF (Figure 2C).These results suggested that deguelin suppresses site specific tyrosine phosphorylation of EGFR.

### Effect of Deguelin on p-ERK/ERK

We examined ERK/p-ERK protein expression in MDA-MB-231 cells incubated in serum free media containing either EGF, Deguelin or Deguelin +EGF. Protein lysates were examined by western blot analysis. MDA-MB-231 cells show constitutively high expression of p-ERK/ERK even when the cells were incubated in the serum free media without any growth factor added (at 2 h and 24 h). Addition of EGF had no effect on ERK/p-ERK expression. However at 24 h, Deguelin treatment reduced the expression of p-ERK and did not change total ERK levels irrespective of presence/absence of EGF in the media (Figure 2D). These results were further confirmed by immunofluorescence examination in the cells treated with Deguelin (with and without EGF) for 24 h. P-ERK expression was localized to both cytosol and nucleus in control cells, Deguelin treatment reduced the p-ERK levels, EGF treatment showed enhanced expression in the nucleus, which was significantly reduced when Deguelin was added to the media (Figure 2D). Similarly, Deguelin treated cells showed reduced expression of Survivin as determined by western blot and immunofluorescence examinations (Figure 2D).

### Effect of Deguelin is Dose Dependent

We examined whether the effect of Deguelin on p-ERK expression is dose dependent. Cells were incubated serum free media with varying concentration of Deguelin. Protein lysates were subjected to western blot analyses. Deguelin inhibited p-ERK and its downstream target p-STAT-3 and c-Myc expression in a dose dependent manner (Figure 2E).

### Deguelin Effect is also seen in the Presence of Serum in Culture Media

**Figure 3:**
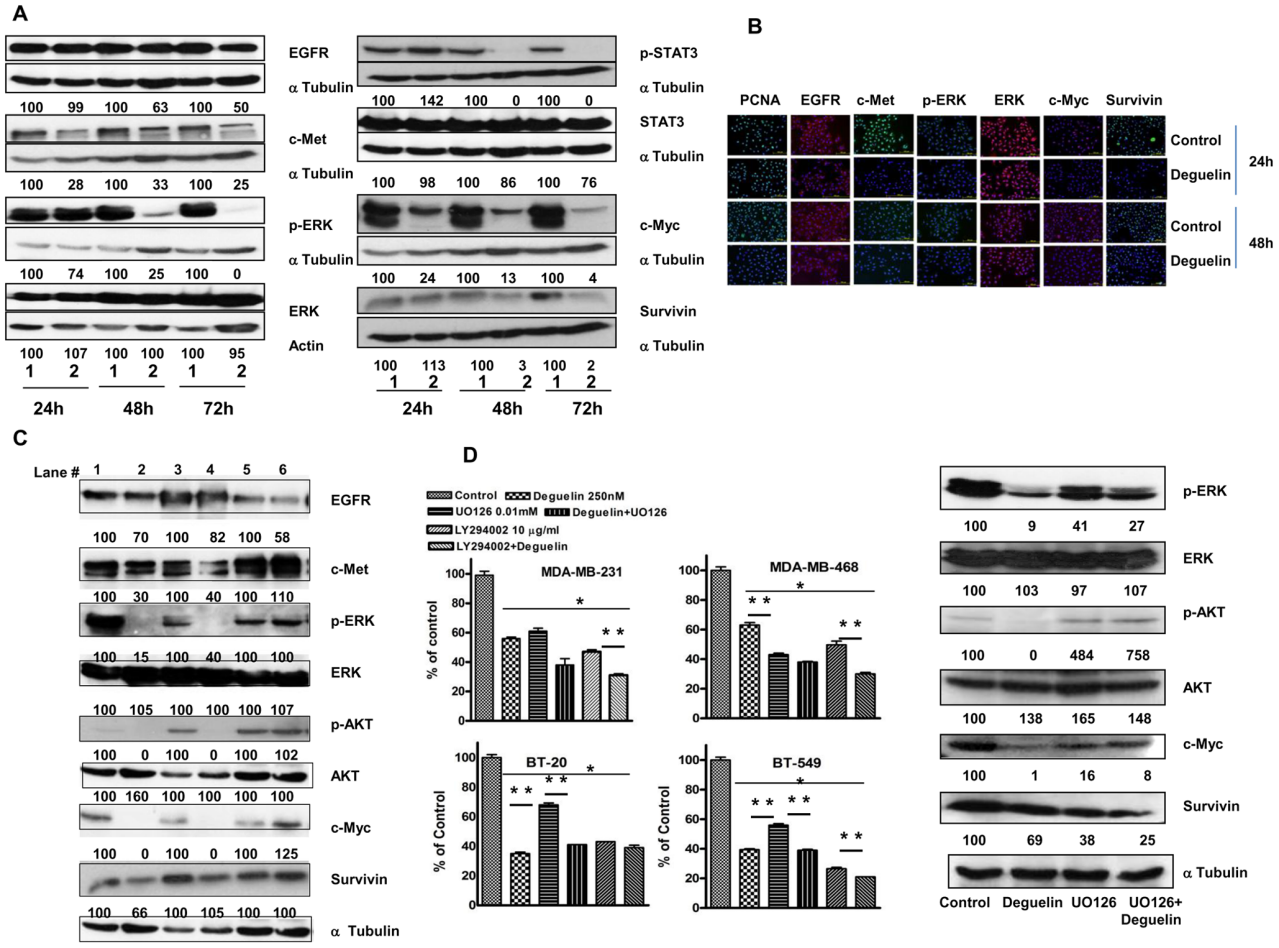
**Effect of Deguelin on EGFR, c-Met and other downstream target proteins in MDA MB 231 cells.** A. Effects of deguelin on the signaling molecules (EGFR, c-MET, p-ERK, ERK, p-STAT3, STAT3, c-MYC, Survivin) in the presence of 10% FBS. MDA-MB-231 cells were treated with indicated concentration of Deguelin or vehicle alone for 24–72 h in the media containing 10% serum. The cell lysates were processed for western blots. Representative western blots are shown. Note: Same Western blot membrane was processed for multiple proteins, this is reflected by same banding pattern of loading control protein. Numbers under each band show % of control of normalized (specific protein/loading control protein) pixel intensity. Values in the respective control were considered as 100%. 1 = Control; 2 = Deguelin (250 nM) treated. B. Effect of Deguelin on expression of various proteins in MDA-MB-231 cells. Cells were plated on glass coverslips, treated with Deguelin (250 nM) or vehicle only for 24, 48 h, serum containing media was used, immunofluorescence analysis was done as described above. Red/green fluorescence = specific protein expression as labeled, blue = DAPI staining for nuclei. C. Effect of vehicle or Deguelin in other breast cancer cell lines. Cells (MDA-MB-231-lanes 1,2; MDA-MB-468 lanes 3,4 and BT-549 lanes 5,6) were treated with vehicle only (lanes 1,3,5) or 250 nM Deguelin (lanes 2,4,6) for 48 h (in culture media with 10% serum), at the end of incubation cell lysates were prepared and subjected to western blot analysis for EGFR, c-Met, p-ERK, ERK, p-AKT, AKT, c-Myc and Survivin). MDA-MB-231 was included as a positive control. D. Effect of ERK UO-126) and PI-3K (LY294002) inhibitors on growth of MDA-MB-231, MDA-MB-468, BT-549 and BT-20 cells: Cells were plated in six well plates, allowed to attach to the well for 24 h and then treated for 72 h with vehicle only, Deguelin (250 nM), UO126 (10 µM), UO126+Deguelin(250 nM), LY294002 (10 µM) and LY294002+Deguelin (250 nM). The culture media contained 10% serum. Cells were counted at termination. Data represent mean± SE of % of control cells. Number of cells in vehicle treated control was considered as 100%. * indicates significant difference between control and treatment, ** indicates significant difference between two groups shown (p<0.05). Western blot shows changes in p-ERK, ERK, p-AKT, AKT, c-Myc and Survivin levels in MDA-MB-231 cells treated with vehicle, Deguelin (250 nM), UO126 (10 µM) and UO126(10 µM) +Deguelin (250 nM). Representative western pattern for each protein is shown.

We examined the effect of Deguelin on molecular markers of EGFR and c-Met signaling cascade (EGFR, c-Met, p-ERK, p-STAT 3, c-Myc, Survivin) in MDA-MB-231 cells incubated in the presence of serum in the culture media. Western blot analysis suggested that similar to those results obtained in these cells in serum free media Deguelin down regulated p-ERK, p STAT-2, c-Met, c-Myc and Survivin in a time dependent manner (Figure 3A) These results were further supported by immunofluorescence examination (Figure 3B). We also examined the effect of Deguelin treatment in other cell lines on the key protein molecules found to be affected in MDA-MB-231 as described above. Interestingly, western blot analysis indicated that Deguelin reduced the expression of p-ERK and p-AKT in MDA-MB-468 cells also but not in other two cell lines (Figure 3C). In order to confirm that the effect of deguelin is mediated by interacting with ERK signaling, we examined the effects of Deguelin, an ERK inhibitor UO126 and combination of both in all four cell lines following 48 h incubation with indicated agent. In MDA-MB-231 and BT-549 and BT-20 Deguelin inhibited cell proliferation in the range of 45 to 65%. Similarly U0126 was also as effective as deguelin. On the other hand in MDA-MB-468 cells UO126 was more effective in inhibiting cell growth as compared to Deguelin. These results indicated that the effects of deguelin amongst TNBC cells are variable. Combination treatment of both ERK inhibitor UO126 and Deguelin was significantly more effective in all except in MDA-MB-468 cells. Similarly we also examined growth inhibitory effect of PI-3 K/AKT inhibitor LY294002 alone or in combination with Deguelin. Treatment with LY294002 significantly (P<0.001) inhibited growth of all cell lines studied, moreover combination treatment with Deguelin+ LY294002 further enhanced the growth inhibitory effect in all except in BT-20 cells (Figure 3D). These results suggested that deguelin mediates its action not only by PI3K/AKT pathway but multiple pathways.

To further confirm our findings that Deguelin effect is probably due to inhibition of multiple signaling pathways we performed western blot analysis in MDA-MB-231 cells treated for 48 h with Deguelin, UO126 and combination of both. Our results clearly showed that Deguelin reduced both p-AKT, p-ERK and their downstream targets c-Myc and Survivin; UO126 had effect on only p-ERK levels and not on p-AKT levels. Combination treatment with both did not show any additive or synergistic effects on any of the parameters studied except on Survivin expression (Figure 3D).

### Deguelin Inhibits in vivo Growth of MDA-MB-231 Cells

**Figure 4:**
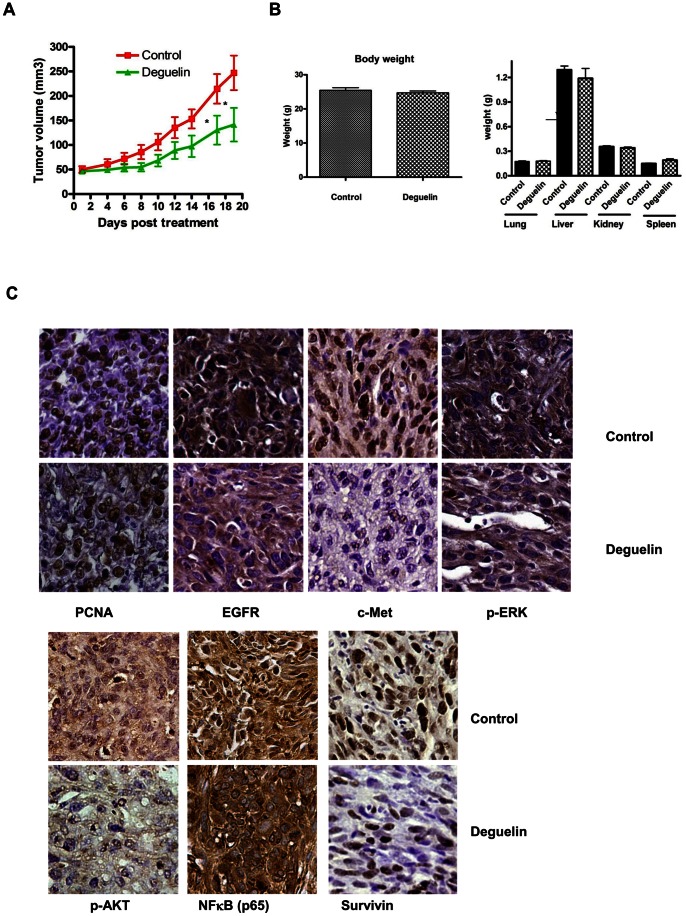
Effect of Deguelin on in vivo growth of MDA-MB-231 cells. A. MDA-MB-231 cells (3 million cells/animal) were injected s.c. in the dorsal flank of 6–7 weeks old female athymic mice. Animals were monitored for the growth of tumor at the injection sites. Once the tumor reached 50 mm^3^ animals received vehicle only or Deguelin (4 mg/kg bodyweight) daily by i.p. route. Tumor size was determined twice/weekly using calipers. Data are shown as mean±SE tumor volume (mm^3^) in each group consisting of n = 10 animals. * indicates statistical difference (p<0.05) between control and treatment group at the indicated time. B. Effect of Deguelin on body visceral organ weights: Animals treated with vehicle/Deguelin as described above, at termination body weights were recorded, lung, liver, kidney and spleens were excised at termination of experiment and weighed. Data in the bar graph represent mean+SE (weights in g) of 10 values in each group. * indicates significant difference between respective control and treatment. C. Immunohistochemical staining of PCNA, EGFR, c-Met, p-ERK, p-AKT, NF-KB (p65) and Survivin proteins in MDA-MB-231 xenografts: Paraffin embedded sections of MDa-MB-231 xenografts from vehicle and Deguelin treated animals were processed for immunohistochemical staining of indicated protein. Brown = specific protein staining, Blue =  counterstaining of nuclei with hematoxylin. Representative images taken at 40× objective are shown.

We examined the effect of Deguelin on in vivo growth of MDA-MB-231 cells transplanted in to 6–7 weeks old athymic mice. MDA-MB-231 cells (3 million cells/mouse) were injected into dorsal flank; animals were monitored for the growth of palpable tumor at the site of injection. Once the tumor reached approximately 50 mm^3^ in volume animals received either vehicle as control or Deguelin at 2 mg and 4 mg (per kg body weight) daily by i.p. injection. In control group 8/10 animals showed continued growth of tumor, two animals did not show any growth as compared to that at the time of treatment initiation. At termination mean tumor volume in control group was 247.6±35.21 cm. In Deguelin (2 mg dose) treated animals one/10 animals failed to show any change in the tumor size during the course of study. Mean tumor volume in this group at termination was 162±32 cm^3^. In animals receiving 4 mg Deguelin one animal failed show change in the tumor volume, in two animals tumor completely disappeared, mean tumor volume at termination was 119±32 (cm^3^)Growth response of MDA-MB-231 cells in animals treated with vehicle and Deguelin (4 mg/kg body weight) is shown in Figure 4A. Statistical analysis (paired t test) suggested that Deguelin (both at 2 and 4 mg dose) treated animals had significantly (P<0.004) smaller tumors as compared to vehicle treated controls. In order to determine whether deguelin is absorbed systemically and accumulated in the tissues, blood, tumors, livers and lungs were collected 2 hours after the last 4 mg/kg body weight deguelin injection in tumor bearing athymic mice. Results showed that there was 3.54±0.4 ng/ml (n = 3) deguelin in the plasma suggesting that deguelin was systemically available. Similarly there was accumulation of 63.4±13.8 ng/g tumor, 17.3±2.8 ng/g lung (n = 3) and 16.3 and 24.1 ng/g liver (n = 2) was found. These results indicated that treatment with deguelin results in tissue absorption and accumulation.

To assess the toxicity associated with the drug treatment, body weights were monitored throughout the course of the study. Body weights were not significantly different in control as compared to Deguelin treated animals. Similarly organ weights of liver, lung and kidney at the time of termination were similar in control and deguelin treated animals, however spleen weight was higher (p<0.01)in Deguelin treated animals than in control vehicle treated group (Figure 4B).

### Down Regulation of EGFR, p-AKT, p-ERK, c-met, NF-κB and Survivin by Deguelin in Xenografts

To confirm the results obtained in vitro in MDA-MB-231 cell line, we examined expression of target molecules in xenografts. Immunohistochemical staining (4 mg/kg body weight) indicated that nuclear PCNA, EGFR (both cytoplasmic and membrane), p-ERK c-Met (nuclear), p-AKT and cytoplasmic and nuclear NF-κB, nuclear Survivin protein expressions were relatively reduced in xenografts obtained from animals treated with Deguelin (4 mg/kg body weight) as compared to those from vehicle treated animals (Figure 4C).

## Discussion

Amongst different molecular subtypes, for patients with tumors lacking ER, PR and Her-2 (classified as triple negative), often systemic chemotherapy is the only option [1]. TNBCs more frequently express BRCA1 mutation. TNBCs occur most frequently in younger and African American women and are quite aggressive. Initially patients with TNBC respond to conventional chemotherapy but relapse rate is higher than other breast cancers [1]. Eventhough TNBCs do not express steroid hormone receptor and Her-2, overexpression of other growth factor receptors such as EGFR and c-Met is quite common [15], [16]. Signaling through both EGFR and c-met that are also receptor tyrosine kinases, promotes cell survival and proliferation by activating various downstream signaling pathways, more specifically PI-3K/AKT/ERK. EGFR/c-Met also activate many downstream proteins including STAT3. STAT3 is activated by Tyr 705 phosphorylation, which triggers the dimerization, nuclear accumulation and further phosphorylation by p- ERK1/2. DNA binding of p-STAT3 facilitates its transcriptional activity. It is believed that P-STAT3 acts as an oncogene through transcriptional activation of target genes to enhance proliferation (eg. c-Myc), angiogenesis (VEGF, ADM, and ANGPTL4), invasion (FGA, FGB, CTSB, and SERPINE2), and suppression of apoptosis (BCL-xL, BCL-2, MCL-1, and BIRC5). In addition, P-STAT3 also stimulates its own transcription causing an increase in STAT3 protein expression [17], [18], [19]. c-Myc is also a transcriptional factor, which regulates expression of Survivin, a major antiapoptotic protein. MAPK further phosphorylates c-Myc and then translocates to the nucleus where it activates or/suppresses its target genes. c-Myc functions as a transcriptional activator and regulates expression of Survivin protein [17]. Survivin expression is present during embryonic development but in most healthy differentiated organs it is not detectable. However, in malignant tumors Survivin overexpression is observed quite frequently [20], [21]. Survivin is known to block the apoptotic cell death by blocking the activity of several caspases [22]. In general increased Survivin expression in tumors reflects an unfavorable prognosis, it correlates with decreased overall survival, increased risk of recurrence, loco regional lymph node invasion and distance metastasis [23].

In this study we demonstrate that Deguelin targets multiple signaling pathways described above and thus inhibit cell proliferation and survival of TNBC cells. Deguelin is a plant derived product and has been reported to inhibit signaling molecules of AKT pathway. Deguelin treatment in vitro has shown promising anticancer effect in various breast cancer cell lines [8], [24]. We studied the effects of Deguelin on the growth of 4 different TNBC cell-lines, namely MDA-MB-231, BT-20, MDA-MB-468 and BT-549. All these four cell lines overexpress EGFR and c-Met. Deguelin in a time and dose dependent manner inhibited growth of all four cell lines studies. As EGFR is the most common receptor expressed in these cell lines we examined whether Deguelin effect is mediated through EGFR modulation. EGF (a known ligand) subsequent to the binding to EGFR induces receptor dimerization, activates kinase domain of receptor and then recruits and phosphorylates number of substrate molecules which leads to intracellular signaling leading to cell proliferation, cell survival or migration. In this study we first tested whether Deguelin will affect cell growth in the presence of EGF. In MDA-MB-231 cells, EGF showed no growth stimulatory effect when incubated for 72 h (1 or 20 ng) suggesting that these cells are insensitive to growth stimulation by EGF. Our results were similar to those reported by others where MDA-MB-231 has been shown nonresponsive to EGF treatment [25]. Interestingly Deguelin was effective in inhibiting cell growth when incubated in the presence or absence of high dose (20 ng) of EGF but not when incubated with low dose(1 ng EGF). The later findings prompted us to look into changes in EGFR expression following EGF and Deguelin or EGF+Deguelin treatments. Immunofluorescence results suggested that Deguelin treatment alone reduced cell surface expression of EGFR. In the presence of low EGF (1 ng) concentration, Deguelin failed to induce changes in cell surface EGFR expression. The cell growth data combined with EGFR expression result described here indicate that Deguelin is effective in the absence of low levels of active cell surface EGFR. Results on western blot analysis further indicated that Deguelin treatment alone or with 20 ng EGF reduced total EGFR, as well as phosphorylated EGFR. Down regulation of EGFR reduced the expression of p-AKT. In addition, at 24 h basal Phospho1045 EGFR band was detected in all the treatments irrespective of presence of EGF or Deguelin. This is unexpected although in many cancer cell lines with either endogenous or exogenously introduced K-ras mutation, production of erbB1 ligands, mainly TGFα and AREG, is up regulated [26], [27]. MDA-MB-231 cells are known to harbor point mutation at codon 13 in the K-ras oncogene [28], [29]. Thus these growth factors secreted from the cells could regulate basal levels of EGFR internalization, recycling and degradation. Deguelin treatment (alone or in combination to EGF) reduced the phospho tyrosine 1045 EGFR. The reduced phospho EGFR following Deguelin treatment observed in these cells may be a reflection of reduced total cell surface EGFR expression and not due to increased degradation. Collectively the results on immunofluorescence and western blot analyses of EGFR indicated that Deguelin down regulates cell surface receptors, and growth inhibitory effect of Deguelin is more pronounced when cells express low EGFR. Thus, the effect of Deguelin does not appear to target EGFR degradation pathway. We examined the effect of Deguelin on c-Met/EGFR -ERK/p-ERK –c-Myc-Survivin expression in the presence/absence of EGF or serum in the culture media. The western blot analyses and immunofluorescence results clearly showed that Deguelin treatment reduced c-Met, EGFR, p-ERK, p-STAT3, c-Myc and Survivin in MDA-MB-231 cells irrespective of presence/absence of EGF or serum in the culture media. The Deguelin effect was dose and time dependent. As expected and reported by others MDA-MB-231 cells expressed constitutively high p-ERK levels in control, Consistent with the report in the literature, no additional effect on of EGF treatment on p-ERK levels was noticed [25].

We compared the growth inhibitory effects of Deguelin and known inhibitors of ERK (UO126) and PI-3k/AKT(LY294002). All the treatments including UO126, LY294002 and Deguelin inhibited growth of breast cancer cells. Both Deguelin and UO126 inhibited p-ERK levels and its downstream targets (c-Myc, survivin) but p-AKT levels were reduced only by Deguelin. Interestingly combination treatment had greater growth inhibitory effect than either agent alone but p-ERK/p-AKT/c-Myc expression failed to correlate with growth data. Interestingly, p -ERK levels were slightly further reduced following combination treatment as compared to UO126 or deguelin treatment alone. These results suggested that possibly both UO126 and Deguelin inhibit p-ERK partially through the same pathway. Another interesting observation was that p-AKT levels were reduced only following Deguelin treatment but not by UO126 or combination of both. It is quite possible that inhibition of p-ERK enhances the p-AKT levels due to cross regulation of p-ERK and p-AKT as suggested others [30]. Prolonged treatment of cancer cells, expressing increased Ras mutation and c-Met amplification, with ERK inhibitors results in increased p-AKT levels.

The effect of Deguelin was further confirmed in vivo in athymic mice. Deguelin (4 mg/kg body weight) treatment given daily by i.p. route significantly inhibited growth of MDA-MB-231 cells transplanted s.c. in athymic mice without causing any serious toxic effects. Results also showed that deguelin in fact is absorbed and accumulated in the tissues including tumor tissue. Immunohistochemical staining of biomarkers in the xenograft confirmed the in vitro findings and showed that Deguelin down regulates EGFR-p-AKT, c-Met-p-ERK, c-Myc and Survivin pathway. We also observed down regulation of p-AKT and NF-κB in tumors from deguelin treated animals. These results are similar to those obtained in vitro in cells and also those reported by others [31], [32]. However, we believe that reduced tumor growth in vivo is unlikely due to reduced c-Met expression. It is now known that mouse derived HGF has lower affinity for human c-met receptor. Thus endogenous host (mouse cells) derived HGF may not stimulate the growth of human breast cancer cells transplanted in athymic mice [33]. Transgenic animals producing human HGF are reported to show that c-Met is involved in in vivo tumor growth [34]. Therefore based on the human c-Met incompatibility with mouse HGF we believe that growth inhibitory effect of deguelin observed in our study may not be associated directly with c-met down regulation. Second scenario is that MDA-MB-231 cells have constitutively active expression of c-Met [35]. Thus effect of c-Met expression is ligand independent. In other words, down regulation of c-met may affect EGFR and its downstream targets as both these receptors are known cross talk with each other and regulate their activation [36]. Thus down regulation of c-Met down regulates EGFR and its downstream targets leading to reduced cell survival and cell growth in vivo. We observed reduced expression of EGFR, p-AKT, NF-κB and also survivin expression in tumors from deguelin treated animals. Collectively our in vivo data suggest that probably inhibition of EGFR expression following Deguelin treatment is associated with reduced tumorigenicity. EGFR is also known to activate STAT-3 and thus its downstream target genes involved in cell proliferation and survival (such as c-Myc and survivin). Many EGFR inhibitors such as ZD1839 has been shown to inhibit in vivo growth of MDA-MB-231 cells [37]. In addition to growth factors cytokines (IL-6 and IL-8) are also known to regulate cell proliferation by modulating STAT-3 expression and thereby its target genes (c-Myc). These cytokines are NF-κB response genes [38]. Deguelin through inhibition of NF-κB activation [31], [32] inhibits IL-8 expression in colon cancer cells [39]. IL-6 gene transcription is also regulated by STAT-3 [40] although effect of deguelin on IL-6 expression is still not known. Thus in addition to EGFR, role of other cytokines (IL-6 or IL-8) in deguelin mediated growth inhibitory action could not be ruled out. We believe that deguelin inhibits EGFR/c-Met pathways and reduces p-AKT which in turn results in reduced nuclear NF-κB expression. Reduced NF-KB additionally may reduce IL-6 mRNA expression, which in turn could inhibit IL-6 protein expression and reduce STAT-3 activation and affect its target genes (such as IL-6 and c-myc) involved in cell proliferation.

The major concern in developing Deguelin as a possible therapeutic agent is its neurotoxicity reported earlier. Deguelin given continuously by infusion caused Parkinson’s-like symptoms in mice [41], however many reports in last few years indicated that Deguelin given orally/i.p route is well tolerated without causing serious side effects including Parkinson’s like symptoms [42], [43], [44]. In the present study animals receiving Deguelin 4 mg/kg body weight for 20 days showed no signs of toxicity as evident from body weight and other organ weights comparison with those in the control group.

**Figure 5:**
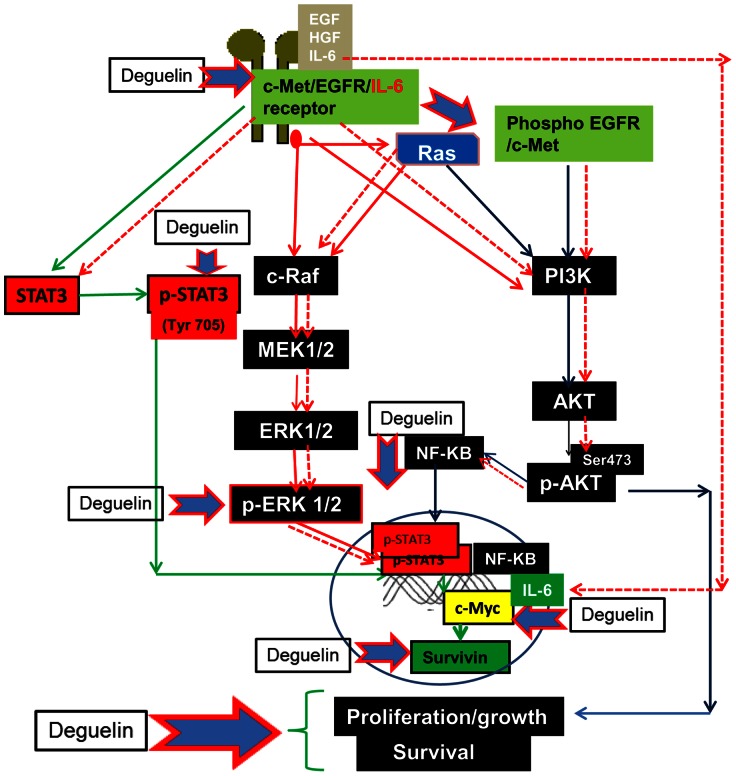
Schematic diagram showing possible mechanism of Deguelin action in triple negative breast cancer cells. Deguelin affects EGFR/c-Met and their downstream target molecules such as p-STAT3, p-ERK, p-AKT, c-Myc and Survivin and there by affect growth/survival of breast cancer cells. Three different pathways affected by Deguelin are shown by different colors; STAT3 (green), AKT (blue), ERK (red). Blue arrows with red outline shows molecules shown to be affected by Deguelin in this study. Red dotted line indicates possible alternative IL-6 mediated pathway which is not explored in this study.

Based on the results in the present study we believe that Deguelin inhibits cell growth in the triple negative breast cancer cells. In selective cell lines Deguelin affects EGFR and c-Met expression, which leads to down regulation of p-AKT, p-ERK, NF-κB and phospho STAT 3 and thereby reduces expression of their downstream targets such as c-Myc and Survivin (Figure 5). Importantly, our data in this study showed that Deguelin is effective in MDA-MB-231 cells. These cells are highly aggressive and harbor K-ras mutation. This finding has a major impact and strongly suggests that Deguelin at non-toxic concentration could be of great therapeutic value not only in TNBC but also other tumor types that are known to harbor K-ras mutation. Thus further clinical evaluation of Deguelin as a single therapeutic agent or in combination with known therapeutic agents for triple negative breast cancer patients is warranted.
